# One Health Approach to *Trypanosoma cruzi*: Serological and Molecular Detection in Owners and Dogs Living on Oceanic Islands and Seashore Mainland of Southern Brazil

**DOI:** 10.3390/tropicalmed10080220

**Published:** 2025-08-02

**Authors:** Júlia Iracema Moura Pacheco, Louise Bach Kmetiuk, Melissa Farias, Gustavo Gonçalves, Aaronson Ramathan Freitas, Leandro Meneguelli Biondo, Cristielin Alves de Paula, Ruana Renostro Delai, Cláudia Turra Pimpão, João Henrique Perotta, Rogério Giuffrida, Vamilton Alvares Santarém, Helio Langoni, Fabiano Borges Figueiredo, Alexander Welker Biondo, Ivan Roque de Barros Filho

**Affiliations:** 1 Department of Veterinary Medicine, Federal University of Paraná (UFPR), Curitiba 80035-050, PR, Brazil; jjuliapacheco12@gmail.com (J.I.M.P.); aaron.ramathan@gmail.com (A.R.F.); perotta@ufpr.br (J.H.P.); ivanbarf@ufpr.br (I.R.d.B.F.); 2 Laboratory of Cell Biology, Carlos Chagas Institute, Oswaldo Cruz Foundation (FIOCRUZ), Curitiba 81310-020, PR, Brazil; louisebachk@gmail.com (L.B.K.); melissapaixao.farias@gmail.com (M.F.); ggustavogonsalves@gmail.com (G.G.); fabiano.figueiredo@fiocruz.br (F.B.F.); 3 Interdisciplinary Graduate Studies, University of British Columbia, Kelowna, BC V1V 1V7, Canada; leandromet@gmail.com (L.M.B.); hlangoni@fmvz.unesp.br (H.L.); 4 Department of Animal Production and Preventive Veterinary Medicine, São Paulo State University (UNESP), Botucatu 18618-681, SP, Brazil; cristielin.alves@unesp.br; 5 Department of Animal Science, School of Life Sciences, Pontifical Catholic University of Paraná (PUCPR), Curitiba 80215-901, PR, Brazil; ruanadelai@ufpr.br (R.R.D.); claudia.pimpao@pucpr.br (C.T.P.); 6 Graduate College in Animal Sciences, University of Western São Paulo (UNOESTE), Presidente Prudente 19050-920, SP, Brazil; rgiuffrida@unoeste.br (R.G.); vamilton@unoeste.br (V.A.S.)

**Keywords:** epidemiology, infection, *Trypanosoma cruzi*, zoonosis

## Abstract

Via a One Health approach, this study concomitantly assessed the susceptibility of humans and dogs to *Trypanosoma cruzi* infections on three islands and in two mainland seashore areas of southern Brazil. Human serum samples were tested using an enzyme-linked immunosorbent assay (ELISA) to detect anti-*T. cruzi* antibodies, while dog serum samples were tested using indirect fluorescent antibodies in an immunofluorescence assay (IFA). Seropositive human and dog individuals were also tested using quantitative polymerase chain reaction (qPCR) in corresponding blood samples. Overall, 2/304 (0.6%) human and 1/292 dog samples tested seropositive for *T. cruzi* by ELISA and IFA, respectively, and these cases were also molecularly positive for *T. cruzi* by qPCR. Although a relatively low positivity rate was observed herein, these cases were likely autochthonous, and the individuals may have been infected as a consequence of isolated events of disturbance in the natural peridomicile areas nearby. Such a disturbance could come in the form of a fire or deforestation event, which can cause stress and parasitemia in wild reservoirs and, consequently, lead to positive triatomines. In conclusion, *T. cruzi* monitoring should always be conducted in suspicious areas to ensure a Chagas disease-free status over time. Further studies should also consider entomological and wildlife surveillance to fully capture the transmission and spread of *T. cruzi* on islands and in seashore mainland areas of Brazil and other endemic countries.

## 1. Introduction

Zoonotic diseases such as toxoplasmosis, brucellosis, leptospirosis, and rickettsioses have been reported in human and dogs living on the oceanic islands of Paraná State, southern Brazil [1,2,3,4]. Such studies have associated zoonosis occurrence and exposure to environmental conditions, including temperature, urban sanitation, drinking water supply, solid waste management, and contact with wildlife species in overlapped environmentally protected areas [1,2,3,4]. These surveys have provided important subsidies for public action regarding the detection, control, monitoring, and prevention of zoonotic diseases in traditional fishing communities located in seashore and island isolated areas.

Chagas disease (CD) is recognized as a neglected tropical disease by the World Health Organization (WHO). It mainly affects socioeconomically vulnerable populations in Brazil, including homeless individuals [5], Bolivian immigrants [6], and rural communities [7]. As the etiological agent of CD, the protozoan *Trypanosoma cruzi* is commonly transmitted through the excreta of triatomine insects, known as kissing bugs, after their blood meal on animal hosts [8]. Oral transmission is currently recognized as the main transmission form of *T. cruzi* in Brazil, which occurs through the ingestion of food or drink contaminated with triatomine feces [9].

In addition, *T. cruzi* may be transmitted vertically (congenital), through a blood transfusion or organ transplantation [10]. *Panstrongylus megistus* is considered one of the main *T. cruzi* vectors in Brazil, with a nationwide distribution [11]. A previous triatomine occurrence survey suggested a higher risk of *P. megistus* occurrence in areas with warmer temperatures (26–29 °C) and a higher relative humidity (70%) [12], which favors the occurrence of this vector on oceanic islands and in coastal mainland areas of southern Brazil. The first stage of infection is acute and usually asymptomatic, and the second is chronic with indeterminate digestive and/or cardiac involvement [8]. *T. cruzi* is maintained by wildlife and triatomine transmission cycles in natural environments [13], and it may also infect domestic animals such as dogs, which can act as sentinels for the occurrence of the disease [14]. The proximity of households to wild environments, combined with the presence of traditional communities with low income and limited access to healthcare services, has created a scenario of vulnerability—favoring less evident, yet still relevant, vector-borne transmission cycles—from a public health perspective [15,16]. Thus, locations near natural areas have been more exposed to the sylvatic cycle of *T. cruzi* and may require constant surveillance and monitoring.

Although *T. cruzi* may occur along the seashore and on islands in southern Brazil, no study to date has concomitantly surveyed exposed owners and dogs. Accordingly, the aim of the present study was to assess the presence of anti-*T. cruzi* antibodies and perform a *T. cruzi* molecular detection assay, testing for the presence of *T. cruzi* in the blood samples of owners and dogs living on oceanic islands and the seashore mainland of southern Brazil.

## 2. Materials and Methods

### 2.1. Ethics Statement

The study presented herein was approved by the Animal Use Ethics Committee of the Federal University of Paraná (protocol 036/2021, approved on August 2021) and the Human Health Ethics Committee of the Ministry of Health (protocol 84756324.0.0000.0020, updated on 3 December 2024).

### 2.2. Study Design and Area

This study was a cross-sectional survey of humans and dogs from three oceanic islands (Superagui Island, Mel Island, and Peças Island) and two coastal mainland municipalities of Paraná State (Guaraqueçaba and Pontal do Paraná), southern Brazil. The present study’s sampling period was from July 2019 to February 2020.

**Figure 1:**
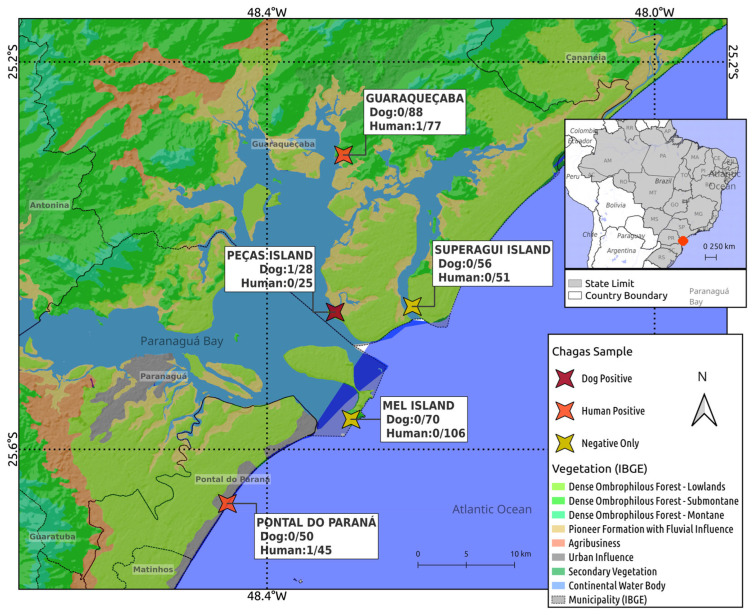
Sampling location and frequency of anti-*T. cruzi* antibodies in humans and dogs from island and seashore mainland areas of southern Brazil.

The study areas were located on the most extended strip of the Atlantic Forest biome, a protected area of Brazil [17]. Mel Island shelters two conservation units, the Ecological Station (81% of the total area) and the State Park (12% of the total area) [18] (Figure 1). Peças Island and Superagui Island are part of the Superagui National Park, two conservation units that include reefs, mangroves, beaches, and endangered species [19]. Pontal do Paraná and Guaraqueçaba are mainland cities that operate as accession routes to the mentioned islands. Guaraqueçaba is a semi-isolated municipality located within three conservation units; access to this area is difficult [19].

### 2.3. Sample Collection

Human participants were sampled after they provided signed consent and completed an epidemiological questionnaire. Blood samples were collected from the human and dog participants during preventive veterinary medicine taskforces. The human blood samples were collected from a cephalic puncture by certified nurses of the Secretary of Health of each municipality. The dog blood samples were collected from a jugular puncture by certified veterinarians of a graduate college, after the owners provided signed consent. The blood samples were collected in EDTA anticoagulant tubes, aliquoted, and stored at −20 °C until processing. Samples were also collected in tubes without an anticoagulant and kept at room temperature (25 °C) until visible clot retraction, centrifuged at 1500 revolutions per minute for five minutes, and separated and kept at −20 °C until processing.

### 2.4. Sample Size and Statistical Analysis

The sample size required to estimate the human prevalence of *T. cruzi* seropositivity was calculated using the epiR package (Version 2.0.85) [20]. An expected prevalence of approximately 5% was assumed, based on a meta-analysis performed in Brazil [21], along with a 95% confidence level and an absolute margin of error of ±2.5%. The analysis indicated that a minimum of 292 individuals would be needed to obtain a reliable prevalence estimative with the specified level of precision and confidence.

To assess the association between epidemiological factors and *T. cruzi* seropositivity in humans, Fisher’s exact test was applied using epiR package [20]. Associations with *p* < 0.05 were considered statistically significant.

### 2.5. Laboratory Analysis

#### 2.5.1. Enzyme-Linked Immunosorbent Assay

The human serum samples were tested to detect anti-*Trypanosoma cruzi* antibodies using a commercial Chagas Test (ELISA Recombinant v.3.0. Kit™, Wiener laboratories, Rosário, Argentina) [22] at the Laboratory of Cell Biology, Carlos Chagas Institute/Oswaldo Cruz Foundation (ICC/Fiocruz, Curitiba, Brasil). The cut-off was calculated by adding an optical density (O.D.) of 0.300 to the average of the negative controls. The positive samples should have values greater than 10% of the cut-off, and the negative samples should have values less than 10% of the cut-off.

#### 2.5.2. Indirect Immunofluorescence Assay

The dog serum samples were tested to detect anti-*Trypanosoma cruzi* antibodies using an indirect immunofluorescence assay (IFA) according to Camargo, 1966 [23]. The same samples were also tested using an IFA for *Leishmania* spp. for comparison purposes due to the possible cross-reaction arising from the close phylogenetic relationship [24]. The serological analyses were carried out at the Laboratory of the Zoonoses Diagnostic Service (SDZ) of the Veterinary Hospital at FMVZ, located at UNESP (São Paulo State University), Botucatu Campus, São Paulo (SP), between July and November 2023.

For the indirect immunofluorescence assay, the immunofluorescence slides were pre-sensibilized with *T. cruzi* antigens (strain Y, maintained by NUPEZO). The samples were tested for IgG antibodies specific to *T. cruzi* using serial dilutions in a microplate at 1:40, 1:80, 1:160, 1:320, and 1:640. Positive and negative control sera from canine samples were also diluted for comparison.

Similarly, for *Leishmania* spp., the *L. major* antigen, maintained by NUPEZO, was used for the IFA. The *L. major* antigen exhibits cross-reactions with all the *Leishmania* spp. circulating in Brazil [24]. The serum samples and positive and negative controls were diluted in PBS (pH of 7.2) at dilutions of 1:40, 1:80, 1:160, 1:320, and 1:640. The samples were considered positive when the titers were equal to or greater than 40.

#### 2.5.3. DNA Extraction and Molecular Analysis

Human and dog seropositive samples were also molecularly tested using qPCR. DNA extraction from whole-blood samples was performed by using the DNeasy Blood & Tissue Kit from QIAGEN (Hilden, Germany), with modifications at the Laboratory of Cell Biology, Carlos Chagas Institute/Fiocruz, and by following the manufacturer’s instructions [25]. The extracted samples were analyzed using the NanoDrop OneC instrument (Thermo Fisher Scientific, Waltham, MA, USA) to confirm the minimum total DNA concentration of 10 ng needed to perform the qPCR.

For the qPCR test, the IBMP Biomol Chagas 2022 kit was used, following the protocol listed in the kit’s instructions [26]. The positive controls provided in the kit were prepared, one with a concentration of 10 copies/µL and the other with 100 copies/µL in RNase-free water (provided in the kit) [26]. The piece of equipment used for the reaction was the Quantstudio™ 5 Real-Time PCR System, and the protocol used was the one established by the kit. The results were read using Quantstudio Design & Analysis Software 2.7.0.

A sample was considered positive for *T. cruzi* if amplification of the *T. cruzi*-specific target (FAM channel) was detected with a Ct (cycle threshold) value between 17 and 45, along with amplification of the internal amplification control (IAC, VIC channel), including cases where only one of the replicates showed amplification. A sample was considered negative for *T. cruzi* if no amplification was observed on the FAM channel but amplification of the IAC (VIC channel) was present.

## 3. Results

The present study assessed the presence of anti-*T. cruzi* antibodies in owners and dogs living on oceanic islands and the seashore mainland, with additional molecular detection in the blood samples of seropositive humans and dogs. A total of 304 individuals and 292 dogs were sampled. Overall, 2/304 (0.6%) owners were seropositive for *T. cruzi* by ELISA, and 1/292 (0.3%) dogs was seropositive by IFA. Both seropositive individuals lived in mainland coastal areas, including a woman from Pontal do Paraná (cut-off value = 0.57), a man in Guaraqueçaba (cut-off value = 2.57), and a positive dog in Peças Island (antibody title = 1:40) (Figure 1; Table 1).

The socioeconomic profiles, according to the questionnaire, indicated that 101/304 participants had a family income equal to or below a single minimum wage. Among them, nine lived in Guaraqueçaba, and eleven were from Pontal do Paraná, including the two positive samples. For the positive individuals, the Pontal do Paraná sample came from a 46-year-old woman with incomplete higher education, residing, but not born, in Pontal do Paraná. This woman was recontacted and provided additional information regarding her background. She reported that her mother was born and raised in the northern Paraná state, an area known for presence of triatomine bugs. She also reported weekly visits to the forest, owning several companion animals (fourteen dogs, five cats, and one guinea pig), drinking treated water, cleaning fruits and vegetables with water, having been bitten by ticks at home several times, and having had both cutaneous larva migrans (*Ancylostoma brasiliensis*) and tungiasis (*Tunga penetrans*) (Supplementary Tables S1 and S2). Overall, 31/304 (10.1%) evaluated individuals reported no contact with dogs, all living in Mel Island. Participants living in the study area reported contact with between 0 and 16 dogs, with a mean of 1.92 ± 1.92 dogs/people.

The positive sample from Guaraqueçaba belonged to an illiterate 80-year-old man with no schooling; he was a resident and local native who reported not visiting the forest, owning two dogs and one cat, washing fruits and vegetables with water, having been bitten by ticks at home, and never having had cutaneous larva migrans but having been contaminated by tungiasis once, with no history of traveling to areas known for active transmission of the disease.

The dogs of both the positive woman and man were seronegative for Chagas disease and *Leishmania* spp. by IFA. All the dogs were seronegative for *Leishmania* spp. The data collected from the questionnaire answered by the dog owner revealed that the positive animal lived in a peridomestic environment, with unrestricted access to the island’s trails, forest, and beaches. The dog also received dry and home food, slept in the open-yard areas, and had the habit of hunting small lizards. In addition, the dog had no history of traveling to areas known for active transmission of the disease.

After the serological analyses, the seropositive individual from Guaraqueçaba and the seropositive dog from Peças Island were contacted, and their blood was resampled, following the ethical and methodological protocol. The individual from Pontal do Paraná was not able to be located for resampling. The previous and resampled blood was subjected to PCR for *T. cruzi* detection. All the samples were positive for *T. cruzi* according to PCR, having the following Ct values: positive human from Guaraqueçaba (Ct values of sample collected in 2019: 38,605; collected in 2024: 42,725); positive human from Pontal do Paraná (Ct value of sample collected in 2019: 36,087); and positive dog from Peças Island (Ct value of sample collected in 2019: 38,029; collected in 2024: 39,025) (Supplementary Table S3). Sequencing of positive samples was not performed.

No associations were observed between seropositivity and the risk factors assessed in the questionnaires for humans (Supplementary Table S4). Statistical analysis was not conducted for dogs because only one dog tested seropositive for *T. cruzi*.

## 4. Discussion

This study reports *T. cruzi* infections in humans and dogs from islands and seashore mainland areas of southern Brazil. The seropositive samples herein were also molecularly positive for *T. cruzi*, suggesting the occurrence of chronic disease [27]. Serological methods are typically used to detect antibodies against *T. cruzi* during the chronic phase of infection with unknown parasitemia [27]. On the other hand, PCR is useful during the acute phase, showing an over 90% sensitivity, with unpredictable applicability during the chronic phase due to the fluctuating parasitemia [27]. The difference in the Ct values between the previous and recollected human and dog samples may indicate some chronicity of the disease, showing a decrease in the parasitic load, as lower values may suggest higher parasitic loads, and higher values may suggest lower parasitic loads [28].

Both ELISA and IFA have been considered highly sensitive tests for the detection of antibodies against *Trypanosoma cruzi*. However, since most commercial kits have been primarily designed for human use, the IFA was selected for testing dog samples. Such a decision was based on the fact that IFA has been routinely used as a daily laboratory technique and has provided advantages including operational simplicity, cost-effectiveness, and reliable results. Indirect immunofluorescence has exhibited high sensitivity, ranging from 90% to 100%, and an approximate specificity of 80% for serum samples, as previously reported [29,30]. For human samples, a commercially available test (Chagatest kit, Wiener, Rosário, Argentina) was used, which demonstrated 100% sensitivity and specificity, according to a recent study [31], which compared various chimeric antigens and commercial assays for the diagnosis of *T. cruzi* infection.

The seroprevalence herein (2/304; 0.6%) was within the nationwide range of 0 to 25.1% and was lower than the average prevalence rate of 4.2% for chronic Chagas disease reported in a meta-analysis of Brazil [21]. Despite the current efforts being made to ensure Chagas disease control, both the acute and chronic phases of Chagas disease remain endemic and mostly underreported in Brazil [32]. In southern Brazil, although *Panstrongylus megistus* and other triatomine vectors of *T. cruzi* have had their presence confirmed [12], only one autochthonous case of acute Chagas disease has been reported in Paraná State since 2018 [33]. It is important to mention that such previous surveys conducted in Paraná State were focused on a Chagas disease profile of reported cases and not on an active surveillance analysis. A retrospective study conducted in the central–northern region of Paraná State reported the clinical profiles of 270 patients with the chronic form of the disease who were affected from 2015 to 2016 [34]. Vector transmission was considered the main form of transmission (91%); the patients were mostly female (64%), most were older than 65 years (60%), and 44% were autochthonous to Paraná State [34]. Another profile study assessing 237 patients from Curitiba, the capital state, from 2007 to 2008 also reported Chagas disease mostly in females (59%), with 60% of the patients having been born and still living in Paraná State [35]. Other studies have reported the occurrence of two variants of *T. cruzi* in the primary and secondary forests of northern and southern Paraná State and a reservoir role for sylvatic species such as *Didelphis marsupialis*, *Didelphis albiventris*, and *Artibeus lituratus* [36,37]. Another study suggested strategic areas to prevent new cases of Chagas disease based on a previous collection of vectors, as well as climatic and landscape variables [12]. That study predicted a higher risk of *T. cruzi* transmission in different areas than those considered in the present study, more specifically in the northwestern, northern, and northeastern areas of Paraná State [12]. Thus, the serological and molecular findings herein may broaden our understanding of the geographical distribution of Chagas disease in southern Brazil.

As previously mentioned, the positive woman reported that her mother was born and raised in endemic areas of Chagas disease, which indicated that her mother may have been infected in the past, possibly asymptomatically, or undiagnosed. In this context, the patient may have acquired Chagas disease through congenital (vertical) transmission, meaning the parasite may have been transmitted from mother to child during pregnancy.

The positive man reported washing fruits and vegetables with water but not drinking treated water. Although no outbreak of Chagas was reported in the present area, oral intake of *T. cruzi* may not be discharged by consumption of contaminated water, drinks, or preparations with triatomine feces [38].

One infected human and the infected dog were confirmed as native to their respective seashore and island, which indicated likely autochthonous transmission in such areas. Thus, although a relatively low prevalence of *T. cruzi* in owners and dogs was observed for the seashore and mainland areas observed herein, the overlap of dwellings with semi-natural areas may predispose vector entering and *T. cruzi* transmission in addition to the sylvatic cycle. This relatively low positivity may also be the consequence of isolated disturbance events in the natural peridomicile areas nearby, such as fires or deforestation events. Such events can cause stress and parasitemia in wild reservoirs and, consequently, spillover to positive triatomines [13]. Due to the lack of a historical presence and as a limitation of the present study, no triatomine trapping was performed during the collection of human and dog samplings.

As previously mentioned, 31/304 (10.1%) individuals reported no contact with dogs, and most participants reported contact with between 0 and 16 dogs, with a mean of 1.92 ± 1.92 dogs/people. Individuals without dog ownership were included as controls, providing potential assessment of seropositivity risk among those with and without contact with dogs using a logistic regression model. Although it was ultimately not possible to model this risk factor, these individuals were retained in the study in accordance with the original study design.

The sample size calculation was based on methods appropriate for continuous epidemiological units, assuming homogeneous transmission dynamics without geographic or social barriers between host populations. However, the present study involves distinct study areas, which may represent separate epidemiological units. Therefore, this assumption may not fully apply to study design. This limitation should be considered when interpreting the representativeness and generalizability of the sample.

As the present study was conducted almost six years ago, the local epidemiological scenario for Chagas disease may have been modified by climate. Previous studies have suggested that higher global temperatures may expand insect vector distribution and Chagas disease transmission into temperate areas [39,40]. Thus, further studies should access humans and their dogs in such areas to provide an update on the epidemiological situation.

The present study, via a One Health approach, aimed to study *T. cruzi* transmission in island and seashore mainland areas of Brazil. Due to the low number of seropositive cases, it was not possible to adequately assess the study areas of infection through either univariate or multivariate analysis. Also, as triatomines have not been reported in such areas, the aim of the present study was primarily to assess human and dog exposure to *T. cruzi*. As six years have passed since this survey, further studies should retest humans and dogs in such areas to update the epidemiological status of Chagas disease spreading.

Only 1 dog out of 292 was positive for *T. cruzi* herein. This dog had unrestricted access to natural areas and hunting habits. In such a scenario, *T. cruzi* may have been transmitted directly by a triatomine, through the hunting and intake of infected wildlife, or through the accidental ingestion of a vector [41]. Such a finding corroborates previous surveys suggesting that dogs can act as sentinels for *T. cruzi* circulation, as a consequence of their transition between domestic and wildlife cycles [13,42,43]. In endemic areas, dogs may act as reservoirs for *T. cruzi* in the domestic cycle due to their proximity to human populations, their high parasitemia without efficient immune control of the infection, and their representation as a blood-meal source, favoring triatomine and pathogen maintenance [44].

## 5. Conclusions

The present study reports positive cases of *T. cruzi* in owners and dogs living on islands and in seashore mainland areas of southern Brazil, improving our understanding of the geographical occurrence of Chagas disease in southern Brazil. Although a relatively low positivity rate was observed herein, the cases were likely autochthonous and may have been the consequence of isolated disturbance events in the natural peridomicile areas nearby, such as a fires or deforestation events. Such events can cause stress and parasitemia in wild reservoirs and, consequently, lead to positive triatomines. Further studies should also consider entomological and wildlife surveillance to fully establish the circulation of *T. cruzi* on oceanic islands and in seashore mainland areas of southern Brazil.

## Figures and Tables

**Table 1:** Total population and sampled individuals of islands and mainland seashore areas in southern Brazil.

Location	Population	Sampling	%
**Seashore mainland**			
Ilha do Mel island	1094	106	9.7
Superagui	700	51	7.3
Peças	350	25	7.1
**Mainland**			
Pontal do Paraná	5000 *	45	0.9
Guaraqueçaba	2182 **	77	3.5
		304	

* only the neighborhood area of the mainland port of Pontal do Paraná City was considered. The city had an estimated 27,915 habitants at the time of sampling. ** only the mainland port of the city’s urban area was considered. Guaraqueçaba city had an overall estimated 7594 inhabitants at the time of sampling.

## Data Availability

The raw data supporting the conclusions of this article will be made available by the authors on request.

## Supplementary Materials

The following supporting information can be downloaded at: https://www.mdpi.com/article/10.3390/tropicalmed10080220/s1, Table S1. Epidemiological information of sampled individuals in islands and seashore mainland areas of southern Brazil; Table S2. Epidemiological information of sampled dogs in islands and seashore mainland areas of southern Brazil; Table S3. Results of the Ct value of qPCR test for the positive samples using the Quantstudio Design & Analysis Software 2.7.0; Table S4. Associated factors for Chagas disease in 304 individuals of islands and mainland seashore areas in southern Brazil.
