# Supplementary material for: One Health Approach to Trypanosoma cruzi: Serological and Molecular Detection in Owners and Dogs Living on Oceanic Islands and Seashore Mainland of Southern Brazil
Source: Trop Med Infect Dis. 2025 Aug 2;10(8):220. doi: 10.3390/tropicalmed10080220 (PMC12390059; doi:10.3390/tropicalmed10080220)
Supplement: Supplementary file 1 [file tropicalmed-10-00220-s001.zip › tropicalmed-3730313-supplementary.pdf]

Supplementary Table S1. Epidemiological information of sampled individuals in islands and seashore mainland areas of southern Brazil.

		Sample location						Total
		GQ[1]	PP[2]	MI[3]	PI[4]	SP[5]	N	
Sex	Male	26	10	49	11	16	112	
	Female	51	35	57	14	35	192	
Educational background	No formal education	2	0	0	0	3	5	1.64%
	Complete elementar school	5	5	13	8	6	37	12.17%
	Incomplete elementar school	10	8	20	2	18	58	19.08%
	Completed high school	26	21	44	6	13	110	36.18%
	Incomplete high school	4	5	6	2	4	21	6.91%
	Completed undergraduate degree	17	2	12	2	2	35	11.51%
	Incomplete undergraduate degree	8	4	9	5	3	29	9.54%
	Postgraduate education	5	0	2	0	2	9	2.96%
City of birth	Local	33	3	23	14	30	103	53.62%
	Other location	44	42	83	11	21	201	46.38%
Number of dogs owned	1 to 2	54	26	96	21	42	239	68.42%
	3 to 4	15	10	6	2	7	40	11,45%
	5 or more	8	9	4	2	2	25	7,16%
Income	No income to 0,5 minimun wage	4	0	1	0	10	15	4,93%

	1 to 1,5 Minimum wage	23	15	46	17	28	129	42,43%
	2 to 2,5 Minimum wage	23	19	25	6	9	82	26,97%
	3 to 3,5 Minimum wage	18	5	13	2	1	39	12,83%
	4 to 5 minimum wage	8	1	11	0	2	22	7,24%
	5+	1	5	10	0	1	17	5,59%
Have ever been bitten by a tick	Yes	42	14	25	8	21	110	36.18%
	No	35	31	81	17	30	194	63.82%
Cutaneous larva migrans	Yes	20	15	48	19	33	135	44,41%
	No	57	30	58	6	18	169	55,59%
Tungiases	Yes	55	35	91	25	50	256	84,21%
	No	22	10	15	0	1	48	15,79%

GQ[1]= Guaraqueçaba city

PP[2] = Pontal do Paraná city

MI[3]= Mel Island

PI[4] = Peças Island

SP[5]= Superagui Island

Supplementary Table S2. Epidemiological information of sampled dogs in islands and seashore mainland areas of southern Brazil.

		Sample location					Total	
		GQ	PP	MI	PI	SI	N	%
Sex	Male	39	20	37	16	37	149	51.02%
	Female	49	30	33	12	19	143	49.03%
Breed	Non-purebreed	68	33	41	23	38	203	69.55%
	Purebreed	20	17	29	5	18	89	30.45%
Level of domiciliation	Domiciled dog	69	37	31	9	34	192	65.75%
	Peridomestic dog	21	9	36	19	20	105	35.95%
	Community dog	4	4	3	0	2	14	4.79%
Supervised access to the trails	Yes	53	4	26	5	25	77	26.37%
	No	35	12	37	10	9	103	35.23%
	No access	38	34	7	13	22	112	38.36%
Supervised access to the beach	Yes	9	11	27	14	33	90	30.82%
	No	7	8	37	14	17	80	27.40%
	No access	72	31	6	2	6	116	39.73%
Supervised access to the forest	Yes	7	5	16	2	17	72	24.66%
	No	23	10	33	11	6	80	27.40%
	No access	58	35	21	15	33	150	51.37%
Hunting behavior	Yes	25	11	28	14	10	95	32.53%
	No	56	21	35	13	44	166	56.85%
	Unknown	7	18	7	1	2	33	11.30%
Ectoparasites	Fleas	55	17	49	26	49	198	67.81%
	Ticks	49	13	18	16	39	134	45.89%
	Does not have	27	32	15	1	5	78	26.71%
Flea and tick control	Yes	57	41	53	22	35	212	72.60%
	No	31	9	17	6	21	80	27.40%

GQ[1]= Guaraqueçaba city

PP[2] = Pontal do Paraná city

MI[3]= Mel Island

PI[4] = Peças Island

SP[5]= Superagui Island

Supplementary Table S3. Results of the Ct value of qPCR test for the positive samples using the QuantstudioDesign&Analysis Software 2.7.0.

Sample ID	Year	Study area	Ct
Positive man	2019	Guaraqueçaba	38.605
Positive woman	2019	Pontal do Paraná	36.087
Positive dog	2019	Peças Island	38.029
Positive man	2024	Guaraqueçaba	42.725
Positive dog	2024	Peças Island	39.025

Supplementary Table S4. Associated factors for Chagas disease in 304 individuals of islands and mainland seashore areas in southern Brazil.

Variables	Positive (%)<br>2 (0.66)	Negative (%)<br>N=302 (99,4)	p.overall
Location:			0.396
Guaraqueçaba	1 (50.0)	76 (25.2)	
Ilha das Peças	0 (0.00)	25 (8.28)	
Ilha do Mel	0 (0.00)	106 (35.1)	
Ponta do Paraná	1 (50.0)	44 (14.6)	
Superagui	0 (0.00)	51 (16.9)	
Gender:			1
Female	1 (50.0)	191 (63.2)	
Male	1 (50.0)	111 (36.8)	
Education level:			1
Incomplete elementary or no education	0 (0.00)	63 (20.9)	
Higher education or more	0 (0.00)	44 (14.6)	
Up to high school	2 (100)	195 (64.6)	
Household income:			1
More than three	0 (0.00)	52 (17.3)	
Between one and three	1 (50.0)	143 (47.5)	
One or less	1 (50.0)	106 (35.2)	
Born in guaraqueçaba:			1
No	1 (50.0)	198 (66.0)	
Yes	1 (50.0)	102 (34.0)	
Dog owner			1
No	0 (0.00)	31 (10.3)	
Yes	2 (100)	271 (89.7)	
Age quartile (years)			0.367
18 to 35	0 (0.00)	81 (26.8)	
35 to 44	1 (50.0)	73 (24.2)	
44 to 56	1 (50.0)	73 (24.2)	
56 to 93	0 (0.00)	75 (24.8)	
